# T‐cell redeployment and intracellular cytokine expression following exercise: effects of exercise intensity and cytomegalovirus infection

**DOI:** 10.14814/phy2.13070

**Published:** 2017-01-13

**Authors:** Emily C. LaVoy, Maryam Hussain, Justin Reed, Hawley Kunz, Mira Pistillo, Austin B. Bigley, Richard J. Simpson

**Affiliations:** ^1^ Department of Health & Human PerformanceUniversity of HoustonHoustonTexas; ^2^ Department of Arts & SciencesUniversity of DelawareNewarkDelaware

**Keywords:** Cytokine, infection, inflammation, lymphocyte, mobilization, physical activity

## Abstract

The magnitude of lymphocytosis following exercise is directly related to exercise intensity. Infection with cytomegalovirus (CMV) also augments lymphocytosis after exercise. It is not known if the enhanced T‐cell response to exercise due to CMV depends on exercise intensity. Furthermore, exercise‐induced changes in T‐cell expression of type I and type II cytokines are thought to be intensity dependent, but direct comparisons are lacking. The aim of this experiment was to determine if CMV affects the exercise‐induced redistribution of T‐cell subsets at varying intensities, and determine the effect of exercise intensity on CD8^+^ T‐cell cytokine expression. Seventeen cyclists (nine CMV seropositive; CMV+) completed three 30 min cycling trials at −5, +5, and +15% of blood lactate threshold (LT). T‐cell subsets in blood and intracellular expression of type I (IL‐2, interferon(IFN)‐*γ*) and type II (IL‐4, IL‐10) cytokines by CD8^+^ T cells pre, post, and 1‐h post‐exercise were assessed by flow cytometry. Independently of CMV, T‐cell subset redistribution was greater after +15%LT compared to −5%LT (*P* < 0.05). Independently of intensity, CMV− mobilized more low‐ (CD27^+^ CD28^+^) and medium‐ (CD27^+^ CD28^−^) differentiated T cells than CMV+, whereas CMV+ mobilized more high (CD27^−^ CD28^−^) differentiated T cells. The numbers of IL‐2+, IFN‐*γ*+, IL‐4+, and IL‐10+ CD8^+^ T cells increased after exercise above LT. Only type I cytokine expression was influenced by exercise intensity (*P* < 0.05). In conclusion, T‐cell redeployment by exercise is directly related to exercise intensity, as are changes in the number of CD8^+^ T‐cells expressing type I cytokines. Although CMV+ mobilized more high‐differentiated T cells than CMV−, this occurred at all intensities. Therefore, the augmenting effect of CMV on T‐cell mobilization is independent of exercise intensity.

## Introduction

Exercise results in a well‐characterized lymphocytosis, leading to substantial changes in the number and composition of lymphocytes subsets in peripheral blood. This response is caused by exercise‐induced increases in shear stress and activation of the sympathetic nervous system and the adrenal medulla (Walsh et al. 2011). Thus, cells with high levels of catecholamine‐binding *β*2‐adrenergic receptors are especially exercise responsive, including highly differentiated antigen‐experienced subsets of CD8^+^ T cells (Steppich et al. 2000; Simpson et al. 2008; Anane et al. 2009; Campbell et al. 2009; Pistillo et al. 2013; Bigley et al. 2014). The magnitude of the leukocytosis is directly related to exercise intensity, where greater mobilizations of immune cells are observed with increasing intensity of exercise (McCarthy and Dale 1988; Shek et al. 1995; Walsh et al. 2011). It is thought that the mobilization of these cytotoxic lymphocyte subtypes in response to acute stress is an adaptive mechanism to enhance immunosurveillance and protect the host under circumstances when wounds or antigen presentation is more likely to occur (Bosch et al. 2005).

Cytomegalovirus (CMV) is a *β*‐herpesvirus that causes intermittently reactivating infection, and is estimated to infect 50–80% of the US population (Staras et al. 2006; Bate et al. 2010). CMV reactivation requires T‐cell responses that lead to persistent clonal expansion and the accumulation of high‐differentiated subsets of T cells (Ouyang et al. 2003b; Pawelec et al. 2004; van de Berg et al. 2010). As these cell types are preferentially mobilized with exercise, CMV infection significantly augments the mobilization of CD8^+^ T cells with exercise (Turner et al. 2010; Lavoy et al. 2014; Spielmann et al. 2014; Simpson et al. 2016). CMV infection also alters the NK‐cell response to exercise, where CMV seropositive donors exhibit a diminished NK‐cell mobilization (Bigley et al. 2012). Interestingly, it has recently been shown that this differential effect of CMV infection on NK‐cell mobilization is only present at exercise above lactate threshold, but not below (Bigley et al. 2015). As the lactate threshold corresponds to the point at which the catecholamines epinephrine and norepinephrine increase exponentially during exercise (Podolin et al. 1991), Bigley et al. (2015) explained the intensity‐dependent effect of CMV by demonstrating that *β*2‐adrenergic receptors on NK cells in CMV+ are less sensitive to the rise in catecholamines. Whether the impact of CMV infection on T‐cell redeployment following exercise depends on exercise intensity remains to be determined.

Exercise also results in changes to immune cell function, including changes in cytokine production (Steensberg et al. 2001; Zaldivar et al. 2006; Lavoy et al. 2013). Cytokines are crucial to the development of pro‐ and anti‐inflammatory immune responses following infection or injury. Cytokine expression by T cells has been used to broadly define two types of immune responses. Type I responses are characterized by the production of interferon (IFN)‐*γ*, whereas type II responses are dominated by IL‐4 (Mosmann and Sad 1996). Moderate exercise is typically reported to increase T‐cell expression of type I cytokines, whereas high‐intensity exercise decreases type I responses in favor of type II (Zhao et al. 2012). These distinct cytokine responses potentially underlie improved immunity following moderate exercise and depressed immunity following vigorous exercise (Steensberg et al. 2001; Walsh et al. 2011). However, this hypothesis is mostly based on the results of separate studies, as little research has compared type I and type II cytokine expression following different intensities of exercise within the same group of participants.

The aim of this study was to examine the impact of latent CMV infection on the exercise‐induced redistribution of T‐cell subsets in response to single bouts of exercise of different intensities. We further aimed to characterize the effect of exercise intensity on the balance of type I and type II T‐cell responses within the same group of healthy adults. We hypothesized that changes in the numbers of T‐cell subsets following exercise would be greater following higher intensity of exercise compared to lower intensity, and that differences in the immune response to exercise between CMV seropositive and CMV seronegative participants would be more pronounced at higher intensities of exercise. Finally, we hypothesized that greater alterations in type I and type II T‐cell responses would be observed following exercise of increasing intensity.

## Methods

### Participants

Seventeen cyclists volunteered for this study. Written informed consent and medical history were obtained from each volunteer after the procedures, benefits, and risks were explained verbally and provided in writing. Adequate training and health status were evaluated with questionnaires, including a cycling history survey, medication use survey, and the ACSM/AHA preexercise readiness questionnaire. Eligibility criteria included participation in road cycling events (i.e., group rides and organized races) at least three times weekly for a minimum period of 12 months. Participants were nonsmokers, did not report the use of any medications known to affect the immune system, and were asked to avoid alcohol, nonprescription drugs, and strenuous exercise for 24 h prior to each laboratory visit. This study conformed to the standards set by the Declaration of Helsinki, and the institutional review board at the University of Houston granted ethical approval for this study.

### Experimental design

Participants visited the laboratory on four occasions and performed four exercise trials, separated by a period of 2 days to 3 weeks. Trial 1 was a lactate threshold test, followed by three 30‐min steady‐state trials at −5, +5, and +15% of blood lactate threshold. All tests were performed at the same time of day, and the steady‐state trials were performed in a randomized, participant‐blinded order. Participants used their personal road bicycle mounted to an indoor cycle ergometer (Computrainer Lab, Racermate, Inc. Seattle, WA). Participants were also screened during the first visit (Trial 1) to recruit equal numbers of cytomegalovirus seropositive (CMV+) and seronegative (CMV−) volunteers. Data were collected from nine CMV+ and eight CMV− participants. Additional data collected in this experimental protocol have been described elsewhere (Bigley et al. 2015; Kunz et al. 2015).

### Blood lactate threshold test

Individual blood lactate threshold (LT) was determined using an incremental discontinuous cycling test consisting of 3‐min incremental stages. Participants were given a 10 min warm‐up and the ergometer was calibrated prior to beginning the protocol. Initial workload and increments were dependent on participants' fitness levels, and ranged from initial workloads of 50–100 W and increments of 20–25 W. Participants maintained consistent revolutions per minute (RPM) throughout the test. Heart rate and breath‐by‐breath metabolic data (VO2, VCO2, ventilation, and respiratory exchange ratio) were measured continuously using a metabolic cart (Quark CPET, Cosmed, Rome, Italy). Ratings of perceived exertion were recorded during the last 30 seconds of each stage (RPE; Borg 6‐20 scale (Borg 1973)). Blood samples drawn from the earlobe at the end of stage were processed immediately in duplicate using an automated precalibrated analyzer (P‐GM7 MicroStat, Analox Instruments, London, UK) to determine blood lactate concentration. The test ended one stage after the blood lactate concentration >4 mmol/L. The power output corresponding to the LT was determined visually using the breakpoint method defined by Weltman (1995).

### Exercise trials and blood sampling

Participants cycled for 30 min at a workload (power output) corresponding to −5, +5, and +15% of their previously identified LT. Participants were blinded to their power output and physiological responses during each trial. Each test began following a 10‐min warm‐up and calibration of the ergometer (set to the same calibration as used for the LT test). Participants used the same RPM as during the LT test. Heart rate and breath‐by‐breath metabolic data were measured continuously, RPE was reported every 5 min, and blood lactate concentration was analyzed every 10 min in capillary blood sampled from the earlobe. Heart rate, VO2, RPE, and blood lactate concentration were averaged over each trial. To ensure hydration, participants were asked to drink plenty of water the day before each trial and 250–350 mL of water the morning of the trial (Sawka et al. 2007).

During Visit 1, participants donated a venous blood sample collected in a serum gel tube prior to the LT test. This was used to determine cytomegalovirus (CMV) IgG antibody titers. During each steady‐state exercise trial, venous blood samples were collected preexercise, postexercise, and 1‐h postexercise in 6‐mL vacutainer tubes treated with ethylene‐diamine‐tetra‐acetic acid (EDTA) (Becton, Dickinson, and Co., Franklin Lakes, NJ). Participants consumed only water until the final blood draw. Blood was processed within 3 h of being drawn.

### Determination of CMV serostatus

Serum samples were obtained by centrifugation and stored at −80°C until analysis. GenWay Biotech (San Diego, CA) ELISA kits were used to detect IgG antibodies to CMV in serum according to manufacturer's directions. Results were read at 450 nm using a 96‐well microplate reader (Molecular Devices, Sunnyvale, CA).

### Flow cytometry

To document exercise‐induced shifts in leukocyte subsets in peripheral blood, whole blood was labeled with monoclonal antibodies and cell phenotypes were assessed on a BD Accuri C6 flow cytometer (BD Accuri, Ann Arbor, MI) as previously described (Lavoy et al. 2014, 2013; Bigley et al. 2014). All antibodies were purchased from eBioscience (San Diego, CA), and included FITC‐conjugated‐anti‐CD28, PE‐conjugated anti‐CD27, PerCP‐Cy5.5‐conjugated anti‐CD4 or anti‐CD8, and APC‐conjugated anti‐CD3. All antibodies were previously titrated to determine optimal dilutions. Single color tubes were used in each assay to account for spectral overall, which was compensated electronically. To document exercise‐induced shifts in cytokine expression by lymphocytes in peripheral blood, peripheral blood mononuclear cells (PBMCs) were isolated using density gradient centrifugation (Histopaque‐1077; Sigma‐Aldrich, St. Louis, MO). PBMCs were incubated at a concentration of 1 × 10^6^ cells/mL in 5% FBS‐RPMI (GIBCO) and Brefeldin A (1 μg/mL; eBioscience Inc) at 37°C with 5% CO_2_ for 12 h. Cells were labeled with FITC‐conjugated anti‐CD3, PE‐conjugated anti‐CD27, and PerCP‐Cy5.5‐conjugated anti‐CD8, fixed in 4% paraformaldehyde, permeabilized in sapononin buffer, and incubated with APC‐conjugated anti‐IL‐2, anti‐IL‐4, or anti‐IFN‐*γ*, or anti‐IL‐10 biotin and streptavidin‐APC.

**Figure 1:**
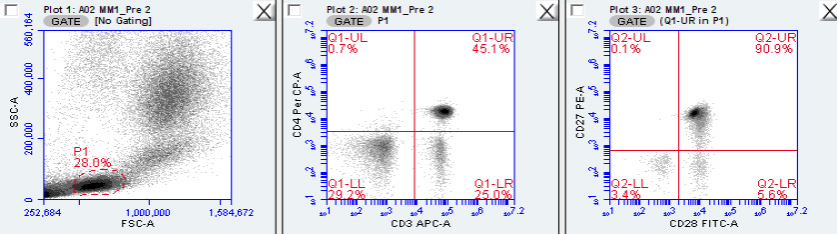
Representative flow cytometry plots from one experimental trial to illustrate gating strategy. In the FSC/SSC‐identified lymphocyte gate (‘P1’, left plot), CD3^+^ CD4^+^ cells (upper right quadrant of middle plot) were further characterized by CD27/CD28 expression (right plot).

In the FSC/SSC‐identified lymphocyte gate, CD3^+^ CD4^+^ and CD3^+^ CD8^+^ cells were further characterized by CD27/CD28 expression: CD27^+^ CD28^+^ were defined as low‐differentiated cells, CD27^+^ CD28^−^ were defined as medium‐differentiated cells, and CD27^−^ CD28^−^ were defined as high‐differentiated cells (Plunkett et al. 2007; van Aalderen et al. 2015). An example of the gating strategy is provided in Figure 1. Cytokine^+^ cells were identified within CD3^+^ cells and CD3^+^ CD8^+^ cells within the lymphocyte gate. Total numbers of each cell subset were determined by multiplying the percentage of all lymphocytes expressing the surface markers of interest by the total lymphocyte count. The lymphocyte counts were measured using a whole blood flow cytometric procedure internally validated against a Mindray BC‐3200 Auto Hematology Analyzer (Nanshan, Shenzhen, PR China)(Bigley et al. 2012; Lavoy et al. 2013).

### Statistical analysis

Normality of data was assessed with the Kolmogorov–Smirnov test and Q–Q plots; data pertaining to the physiological responses and T‐cell subset redistribution met the assumptions of normality. Differences in the physiological responses to the three exercise intensities (−5% LT, +5% LT, and +15% LT) were analyzed using repeated measures analysis of variance tests. Differences in the physiological responses to each exercise session due to CMV serostatus (+, −) were analyzed by Student's T tests. The impact of CMV and exercise intensity on the exercise‐induced redistribution of T‐cell subsets was analyzed using maximum likelihood random intercepts linear mixed models (LMM). This statistical approach is designed for analyzing repeated measures (McCulloch and Searle 2000). Main effects of exercise intensity (−5% LT, +5% LT, and +15% LT), time (pre, post, and post‐1 h), and CMV serostatus (+, −), and factorial interaction effects on the numbers of T‐cell subsets were examined. Significance was set at *P* < 0.05; when significant main effects were found, Sidak post hoc comparisons were used to determine the location of the effect. We also analyzed the main and interaction effects of exercise intensity and CMV on the ingress (change in cell number: postexercise – preexercise) and egress (change in cell number: 1‐h postexercise – postexercise) of each cell subset. T‐cell cytokine expression related to exercise intensity did not meet normality assumptions and could not be log transformed; therefore, these data were analyzed using Wilcoxon Signed‐Ranks Test. This statistical approach is designed to test for the median difference in repeated measures data that do not meet normality assumptions (Gibbons 1993). Median scores of CD8^+^ T cells expressing each cytokine were examined at each level of exercise intensity (−5% LT, +5% LT, and +15% LT). All statistical analyses were performed using the Statistical Package for the Social Sciences (SPSS v23.0, Chicago, IL).

## Results

### Physiological responses to exercise

Physiological and performance data for the three exercise trials are displayed in Table 1. All 17 participants (four females; mean (SD) Age: 30.9 (5.0) year; BMI: 23.1 (1.9) kg m^−2^; Relative power at LT: 2.61 (0.66) W) successfully completed each trial. There were nine CMV+ participants (two female CMV+). CMV+ and CMV− participants did not differ in anthropomorphic measures or in physiological responses to exercise (data not shown, *P* > 0.05).

**Table 1:** Physiological responses to cycling exercise at three intensities (−5% LT, +5% LT, and +15% LT)

Variable	‐5% LT	+5% LT	+15% LT
Power output (watts)	175.6 ± 45.7	195.3 ± 50.6*	213.8 ± 55.9**
Heart rate (bpm)	147.4 ± 12.3	157.8 ± 13.1*	164.5 ± 11.3**
VO_2_ (L/min)	2.7 ± 0.7	3.1 ± 0.7*	3.2 ± 0.8**
Blood lactate (mMol)	1.3 ± 0.7	2.2 ± 0.7*	4.0 ± 1.9**
RPE^1^	11.6 ± 1.3	13.7 ± 1.1*	15.4 ± 1.6**

Data shown are mean ± SD from 17 subjects (four females). Trial difference is indicated by *(relative to −5% LT) and **(relative to −5% LT and +5% LT); *P* < 0.05.

LT, lactate threshold.

^1^Average rating of perceived exertion (6‐20 Borg scale).

### T‐cell redeployment with exercise: effect of time

There were no differences in baseline values for any T‐cell subset between the three exercise trials (*P* > 0.05; data not shown). The numbers of low‐differentiated (CD27^+^ CD28^+^), medium‐differentiated (CD27^+^ CD28^−^), and high‐differentiated (CD27^−^ CD28^−^) CD4^+^ and CD8^+^ T cells were significantly elevated postexercise (*P* < 0.001). The number of low‐ and high‐differentiated CD4^+^ T cells fell below baseline values 1‐h postexercise (*P* < 0.001) (main effect of time; Table 2), whereas the numbers of CD8^+^ T‐cell subsets returned to baseline values 1‐h postexercise (main effect of time; Table 3).

**Table 2:** The numbers of CD4^+^ T cells and CD4^+^ T‐cell subsets present in peripheral blood preexercise, postexercise, and 1‐h postexercise at −5%, +5%, and +15% LT

Cell Subset (cells/*μ*L)		Pre	Post	1‐h Post	Main Effects			Interaction Effects			
					Time	Intensity	CMV	Time × CMV	Time × Intensity	Intensity × CMV	Time x Intensity × CMV
CD4^+^ T Cells			*,**	*	39.81 (0**.000**)	0.113 (0.893)	0.004 (0.953)	0.602 (0.549)	2.50 (0**.046**)	7.05 (0**.001**)	0.317 (0.866)
−5% of LT	CMV +	859.5 ± 129.8	887.3 ± 147.2	676.2 ± 124.2							
	CMV−	686.4 ± 327.0	760.0 ± 272.3	533.1 ± 173.4							
+5% of LT	CMV +	644.7 ± 182.0	816.5 ± 213.3	632.4 ± 155.6							
	CMV−	772.0 ± 460.7	961.4 ± 436.6	624.0 ± 340.7							
+15% of LT	CMV +	671.9 ± 154.0	949.4 ± 199.3	565.5 ± 130.2							
	CMV−	724.8 ± 394.7	1007.3 ± 461.9	578.4 ± 218.5							
CD27^+^ CD28^+^			*,**	*	30.49 (0**.000**)	0.11 (0.896)	0.45 (0.512)	2.34 (0.100)	1.86 (0.121)	6.46 (0**.002**)	0.30 (0.881)
−5% of LT	CMV +	742.3 ± 169.2	745.8 ± 168.9	593.6 ± 138.8							
	CMV−	664.5 ± 318.3	734.2 ± 265.9	515.9 ± 168.9							
+5% of LT	CMV +	562.8 ± 196.7	671.3 ± 216.4	558.9 ± 174.8							
	CMV−	748.4 ± 452.1	923.8 ± 431.6	600.5 ± 333.8							
+15% of LT	CMV +	578.2 ± 185.3	780.7 ± 195.6	514.1 ± 134.6							
	CMV−	697.7 ± 383.3	952.7 ± 443.3	555.1 ± 212.1							
CD27^+^ CD28^−^			*,**		53.39 (0**.000**)	5.81 (0**.004**)	3.76 (0.070)	8.52 (0**.000**)	3.91 (0**.005**)	1.41 (0.249)	0.54 (0.706)
−5% of LT	CMV +	1.4 ± 0.9	1.4 ± 0.8	0.8 ± 5.6							
	CMV−	1.8 ± 1.5	2.9 ± 1.9	0.7 ± 0.0.5							
+5% of LT	CMV +	1.1 ± 0.7	2.3 ± 1.0	1.1 ± 0.7							
	CMV−	1.8 ± 1.5	4.2 ± 2.3	1.2 ± 1.3							
#+15% of LT	CMV +	1.3 ± 0.8	2.7 ± 1.5	0.6 ± 0.5							
	CMV−	1.7 ± 1.3	5.2 ± 3.7	1.7 ± 1.3							
CD27^−^ CD28^−^			*,**	*	33.80 (0**.000**)	1.91 (0.152)	7.71 (0**.013**)	28.45 (0**.000**)	1.26 (0.291)	1.90 (0.154)	1.11 (0.356)
−5% of LT	^CMV +	61.3 ± 78.8	76.6 ± 77.2	34.7 ± 41.2							
	CMV−	1.0 ± 2.1	2.2 ± 3.8	0.5 ± 0.8							
+5% of LT	^CMV +	36.0 ± 39.0	71.3 ± 61.8	29.1 ± 33.2							
	CMV−	0.8 ± 1.4	2.33 ± 2.4	0.6 ± 0.9							
+15% of LT	^CMV +	51.6 ± 68.3	84.10 ± 75.5	21.7 ± 24.6							
	CMV−	1.0 ± 1.3	2.3 ± 2.4	0.8 ± 1.8							

Values are means ± SD; Significance at *P* < 0.05; Time: *differs from pre, ** differs from 1‐h post; Intensity: # differs from ‘−5’, ## differs from ‘+15’; CMV: ^ differs from CMV−; Main and interaction effects are F‐statistic (*P*‐value).

CMV, cytomegalovirus; LT, lactate threshold

Bold values indicate *P* < 0.05.

**Table 3:** The numbers of CD8^+^ T cells and CD8^+^ T‐cell subsets present in peripheral blood preexercise, postexercise, and 1‐h postexercise at −5%, +5%, and +15% LT

Cell Subset (cells/*μ*L)		Pre	Post	1‐h Post	Main effects			Interaction effects			
					Time	Intensity	CMV	Time × CMV	Time × Intensity	Intensity × CMV	Time × Intensity x CMV
CD8^+^ T Cells			*, **		95.20 (0**.000**)	9.53 (0**.000**)	0.004 (0.953)	0.602 (0.549)	2.50 (0**.046**)	7.05 (0**.001**)	0.317 (0.866)
−5% of LT	CMV +	335.7 ± 78.1	463.4 ± 153.2	299.6 ± 33.0							
	CMV−	276.8 ± 110.0	413.3 ± 139.4	250.8 ± 98.2							
#+5% of LT	CMV +	350.9 ± 172.0	254.6 ± 195.1	284.9 ± 38.8							
	CMV−	316.0 ± 102.9	594.5 ± 209.6	310.0 ± 152.3							
#+15% of LT	CMV +	353.9 ± 102.9	645.6 ± 199.6	273.3 ± 70.3							
	CMV−	310.9 ± 129.2	745.1 ± 334.8	283.7 ± 127.2							
CD27^+^ CD28^+^			*, **		45.86 (0**.000**)	8.33 (0**.000**)	0.45 (0.512)	2.34 (0.100)	1.86 (0.121)	6.46 (0**.002**)	0.30 (0.881)
−5% of LT	CMV +	218.3 ± 73.6	259.2 ± 79.6	205.8 ± 53.6							
	CMV−	222.1 ± 114.4	307.3 ± 150.5	216.7 ± 98.6							
#+5% of LT	CMV +	209.6 ± 73.7	285.2 ± 90.8	202.1 ± 44.1							
	CMV−	294.1 ± 169.8	425.5 ± 191.6	261.4 ± 140.5							
#+15% of LT	CMV +	225.5 ± 67.2	340.6 ± 88.2	206.6 ± 61.4							
	CMV−	260.0 ± 129.7	513.5 ± 191.6	244.9 ± 126.2							
CD27^+^ CD28^−^			*, **		75.88 (0**.000**)	6.04 (0**.003**)	3.76 (0.070)	8.52 (0**.000**)	3.91 (0**.005**)	1.41 (0.249)	0.54 (0.706)
−5% of LT	CMV +	36.3 ± 31.5	60.2 ± 37.7	27.8 ± 20.9							
	CMV−	33.1 ± 25.2	62.4 ± 41.6	19.2 ± 13.3							
+5% of LT	CMV +	35.0 ± 33.1	63.4 ± 38.3	24.1 ± 15.2							
	CMV−	33.4 ± 19.3	93.8 ± 58.9	25.8 ± 18.8							
#+15% of LT	CMV +	47.0 ± 48.5	93.0 ± 67.4	24.6 ± 21.5							
	CMV−	29.5 ± 17.4	127.2 ± 89.9	22.6 ± 14.0							
CD27^−^ CD28^−^			*, **		45.89 (0**.000**)	1.40 (0.251)	7.71 (0**.013**)	28.45 (0**.000**)	1.26 (0.291)	1.90 (0.154)	1.11 (0.356)
−5% of LT	^CMV +	74.9 ± 63.3	132.1 ± 90.3	56.6 ± 48.3							
	CMV−	17.3 ± 20.0	35.4 ± 43.4	9.9 ± 13.9							
+5% of LT	^CMV +	65.5 ± 53.9	160.3 ± 136.5	50.2 ± 44.3							
	CMV−	17.7 ± 22.2	60.4 ± 72.6	16.1 ± 19.6							
+15% of LT	^CMV +	74.6 ± 70.2	189.9 ± 158.6	36.2 ± 27.5							
	CMV−	16.6 ± 15.5	87.8 ± 108.4	11.3 ± 10.3							

Values are means ± N; Significance at *P* < 0.05: Time: * differs from pre, ** differs from 1‐h post; Intensity: # differs from ‘−5’, ## differs from ‘+15’; CMV: ^ differs from CMV−; Main and interaction effects are F‐statistic (*P*‐value).

CMV, cytomegalovirus; LT, lactate threshold.

Bold values indicate *P* < 0.05.

### T‐cell redeployment with exercise: effect of intensity

Main effects of intensity were observed for the number of medium‐differentiated CD4^+^ T cells and low‐ and medium‐differentiated CD8^+^ T cells, as well as the total number of CD8^+^ T cells (*P* < 0.01; Tables 2 and 3). Independently of time and CMV, there was a greater number of each of these cell subsets in the +15% LT condition compared to the −5% LT condition. Furthermore, there was a greater number of CD8^+^ T cells and low‐differentiated CD8^+^ T cells in the +5% LT condition than −5% LT.

**Figure 2:**
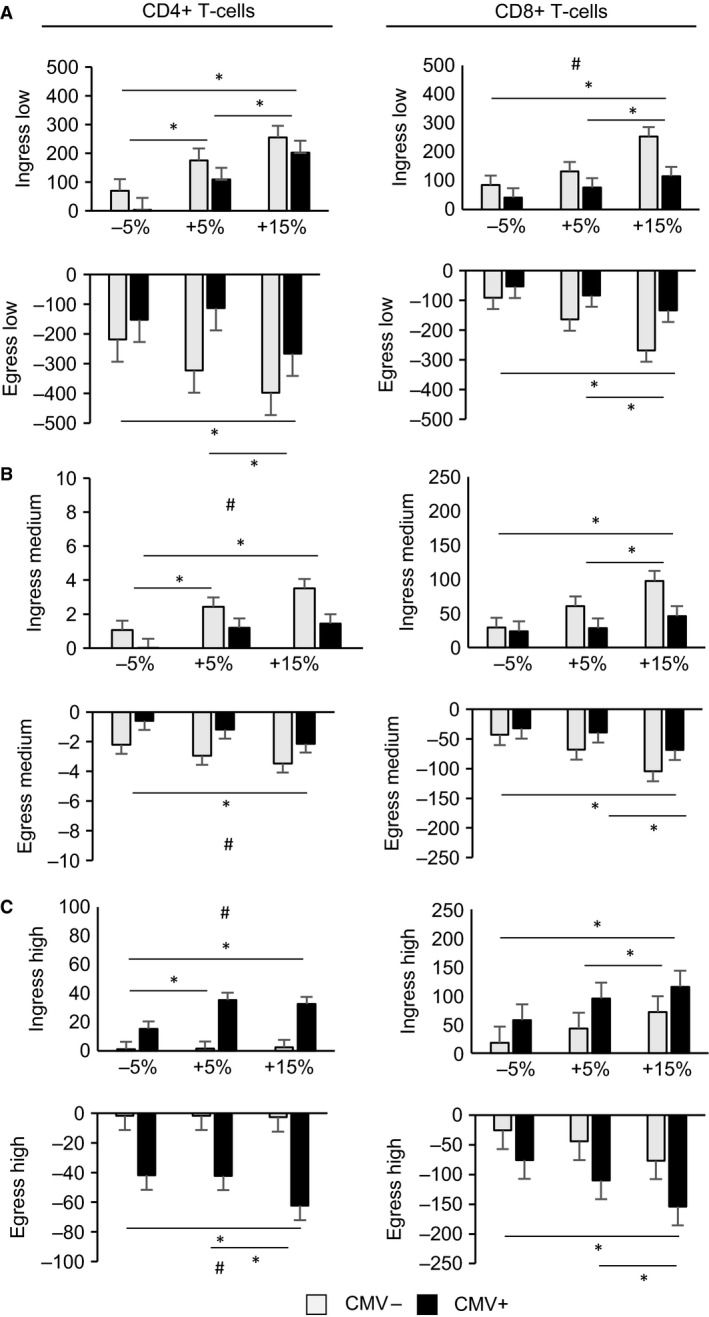
The ingress (postexercise – preexercise) and egress (1‐h postexercise – postexercise) of CD4^+^ and CD8^+^ T‐cell subsets in cytomegalovirus (CMV)+ and CMV− participants following exercise at −5% lactate threshold (LT), +5% LT, and +15% LT. (A) Change in number of low‐differentiated (CD27^+^ CD28^+^) T cells. (B) Change in number of medium‐differentiated (CD27^+^ CD28^−^) T cells, (C) Change in number of high‐differentiated (CD27^−^ CD28^−^) T cells. * Indicates significant difference between intensities; # indicates significant difference between CMV− and CMV+ (*P* < 0.05). Data shown are mean ± SEM.

Independently of CMV, there were significant interaction effects between intensity and time for CD4^+^ and CD8^+^ T‐cell subsets (Tables 2 and 3). These interaction effects were further explored by comparing the ingress and egress of the T‐cell subsets across −5% LT, +5% LT, and +15% LT (Fig. 2). A main effect for intensity was observed for the ingress of low‐ (F_2,32_ = 18.22, *P* < 0.001), medium‐ (F_2,32_ = 6.72, *P* < 0.01), and high‐ (F_2,32_ = 5.64 *P* < 0.01) differentiated CD4^+^ T cells, as well as low‐ (F_2,32_ = 12.09, *P* < 0.001), medium‐ (F_2,32_=13.94, *P* < 0.001), and high‐ (F_2,32_=7.18, *P* < 0.01) differentiated CD8^+^ T cells. A main effect for intensity was also present during the egress of low‐ (F_2,32_ = 6.34, *P* < 0.01), medium‐ (F_2,32_ = 3.89, *P* < 0.05), and high‐ (F_2,32_ = 6.11, *P* < 0.01) differentiated CD4^+^ T cells, as well as low‐ (F_2,32_ = 11.68 *P* < 0.001), medium‐ (F_2,32_ = 16.70, *P* < 0.001), and high‐ (F_2,32_ = 10.84, *P* < 0.001) differentiated CD8^+^ T cells. Post hoc pairwise comparisons revealed that +15% LT led to a significantly greater ingress and egress of the CD4^+^ and CD8^+^ T‐cell subsets compared to exercise at −5% LT (Fig. 2). Significant differences were also observed between −5% LT and +5% LT in the ingress of CD4^+^ T‐cell subsets, and between +5% and +15% in the ingress and egress of CD8^+^ T‐cell subsets (Fig. 2).

### T‐cell redeployment with exercise: effects of CMV

Independently of time or exercise intensity, CMV+ participants had significantly greater numbers of high‐differentiated CD4^+^ and CD8^+^ T cells compared to CMV− (Tables 2 and 3). Independently of exercise intensity, significant interaction effects were observed between CMV and time for medium‐ and high‐differentiated CD4^+^ T cells, and low‐, medium‐, and high‐differentiated CD8^+^ T cells (Tables 2 and 3). These interactions were further explored by contrasting the ingress and egress of T‐cell subsets by CMV serostatus (Fig. 2). CMV− demonstrated a greater ingress of medium‐differentiated CD4^+^ T cells (F_1,16_ = 10.00, *P* < 0.01), and low‐differentiated CD8^+^ T cells (F_1,16_ = 4.83, *P* < 0.05) than CMV+, whereas CMV+ had a greater ingress of high‐differentiated CD4^+^ T cells (F_1,16_ = 18.08, *P* < 0.01). During the recovery from exercise, CMV− displayed a greater egress of medium‐differentiated CD4^+^ T cells (F_1,16_ = 6.21, *P* < 0.05) than CMV+, and CMV+ displayed a greater egress of high‐ (F_1,16_ = 12.78, *P* < 0.01) differentiated CD4^+^ T cells than CMV− (Fig. 2).

### T‐cell redeployment with exercise: interaction effects of intensity and CMV

Significant interactions between intensity and CMV were observed for low‐differentiated CD4^+^ T cells and low‐differentiated CD8^+^ T cells (Tables 2 and 3). Sidak post hoc pairwise comparisons revealed a significant effect of exercise intensity among CMV−: the number of low‐differentiated CD4^+^ T cells and low‐differentiated CD8^+^ T cells present in blood at −5% LT was significantly less than the number of low‐differentiated CD4^+^ T cells and low‐differentiated CD8^+^ T cells present in blood at 5% LT and 15% LT. However, exercise intensity did not significantly impact the number of these subsets among CMV+. There was not a significant interaction among time, CMV serostatus, and exercise intensity for any subset examined (*P* > 0.05).

### T‐cell cytokine expression: effects of time and intensity

T‐cell cytokine expression was transiently increased by exercise. Compared to preexercise, the number of CD8^+^ T‐cells‐expressing type I cytokines (IL‐2: Z = −2.67, *P* < 0.01; IFN‐*γ*:* Z* = −2.31, *P* < 0.05) and type II cytokines (IL‐4: *Z* = −2.67, *P* < 0.01; IL‐10: *Z* = −2.93, *P* < 0.01) was significantly elevated postexercise after +15% LT, as well as after +5% LT (IL‐2: *Z* = −2.93, *P* < 0.01; IFN‐*γ*:* Z* = −2.31, *P* < 0.05; IL‐4: *Z* = −2.67, *P* < 0.01; IL‐10: *Z* = −2.93, *P* < 0.01) (Table 4). Exercise at −5% LT also significantly increased the number of CD8^+^ T‐cells expressing IL‐2 (*Z* = −2.04, *P* < 0.05), IL‐4 (*Z* = −2.40, *P* < 0.05), and IL‐10 (*Z* = −2.49, *P* < 0.05) postexercise compared to preexercise. Compared to preexercise, there were significantly fewer CD8^+^ T‐cells expressing IL‐2 (*Z* = −2.84, *P* < 0.01), IL‐4 (*Z* = −2.31, *P* < 0.05), and IL‐10 (*Z* = −2.22, *P* < 0.05) 1 h postexercise after +15% LT, fewer CD8^+^ T‐cells expressing IL‐2 (*Z* = −2.31, *P* < 0.05), IFN‐*γ* (*Z* = −2.04, *P* < 0.05), and IL‐4 (*Z* = −2.49, *P* < 0.05) 1‐h postexercise after +5% LT, and fewer CD8^+^ T‐cells expressing IL‐2 (*Z* = −2.67, *P* < 0.01) and IFN‐*γ* (*Z* = −2.93, *P* < 0.01) 1‐h postexercise after −5% LT.

**Table 4:** Cytokine‐expressing CD8^+^ T cells (cells/*μ*L) pre‐, post‐, and 1‐h postexercise at three exercise intensities (−5%, +5%, and +15% LT)

Trial	Time	IL‐2	IFN‐*γ*	IL‐4	IL‐10	IFN‐*γ*: IL‐4
−5% LT	Pre	15.58 (13.64)	7.82 (19.86)	10.07 (7.87)	7.16 (13.11)	0.80 (1.62)
	Post	18.50 (46.51)*,**	9.66 (23.59) **	14.53 (26.79)*,**	14.91 (49.31)*,**	0.67 (2.06)
	1‐h post	10.21 (17.97)*	3.29 (16.80)*	7.83 (21.51)	6.68 (12.28)	0.55 (4.14)
+5% LT	Pre	13.28 (24.19)	8.62 (28.95)	8.89 (13.70)	7.89 (16.17)	1.01 (1.25)
	Post	22.73 (45.82)*,**	19.06 (47.41)*,**	19.99 (51.45)*,**	15.45 (46.05)*,**	0.92 (1.82)
	1‐h post	9.12 (16.85)*	6.75 (24.87)*	7.64 (10.31)*	6.75 (17.94)	0.98 (2.86)
+15% LT	Pre	13.09 (17.61)	5.58 (7.72)	10.39 (9.79)	7.09 (13.75)	0.82 (0.89)
	Post	38.74 (46.62)*,**	17.73 (42.95)*,**	18.28 (27.58)*,**	19.15 (29.73)*,**	1.25 (1.48)*
	1‐h post	7.70 (9.37)*	4.32 (14.68)	7.01 (9.92)*	5.52 (15.83)*	0.65 (1.85)

Values are medians (range) for nonparametric tests. Significant main effects indicated by: * differs from pre, ** differs from 1‐h post (*P* < 0.05)

IFN, interferon; LT, lactate threshold.

The ratio of IFN‐*γ*: IL‐4‐expressing CD8^+^ T cells was significantly greater postexercise compared to preexercise in the +15% LT trial (*Z* = −2.13, *P* < 0.05) (Table 4).

**Figure 3:**
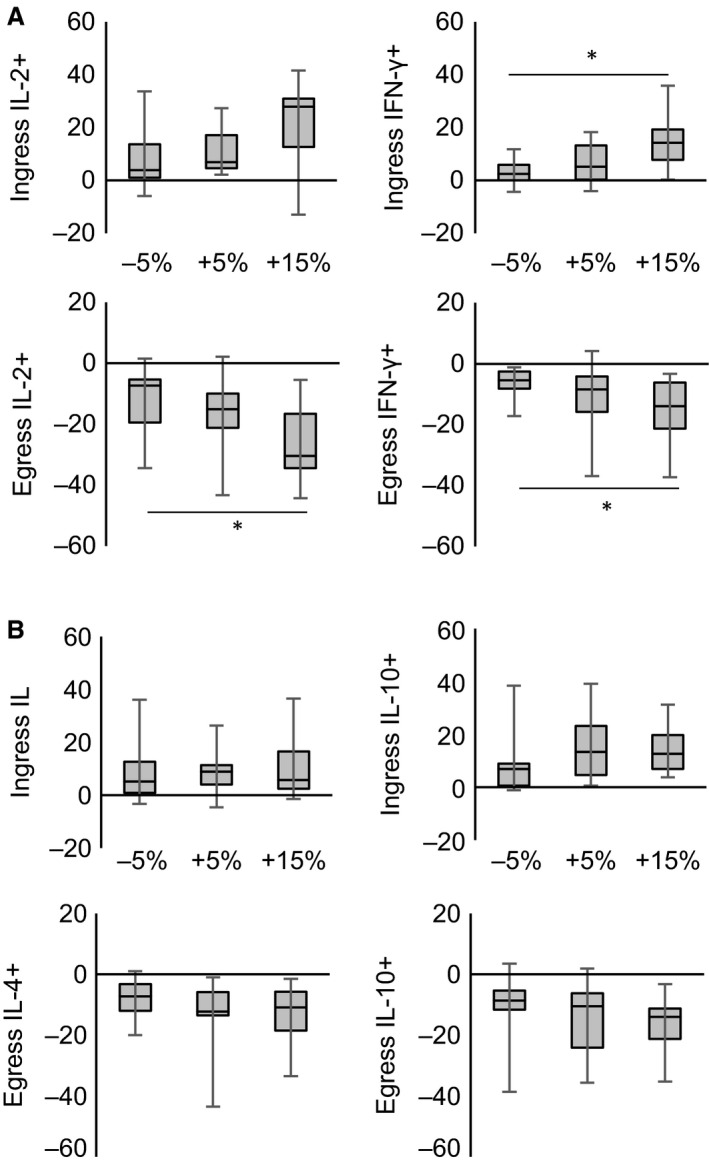
The ingress (postexercise – preexercise) and egress (1‐h postexercise – postexercise) of CD8^+^ T‐cells‐expressing cytokines following exercise at −5% LT, +5% LT, and +15% LT. (A) Change in number of type I cytokine‐expressing CD8^+^ T cells (Δ cells/*μ*L). (B) Change in number of type II cytokine‐expressing CD8^+^ T cells (Δ cells/*μ*L). * Indicates significant difference between intensities (*P* < 0.05). Box plots show median values (solid horizontal line), 75th percentile values (upper box border), 25th percentile values (lower box border), and minimum and maximum values (bars).

The nonparametric tests used for analyses of the cytokine data did not allow us to evaluate potential interaction effects of intensity with time. Instead, we analyzed the effect of intensity on the ingress and egress of cytokine‐expressing CD8^+^ T cells (Fig. 3). The ingress of IFN‐*γ* ^+^ cells was significantly larger during the +15% LT trial compared to −5% LT (*Z* = −2.58, *P* < 0.05). The 1‐h postexercise egress of IL‐2^+^ and IFN‐*γ* ^+^+ cells was greater following +15% LT than −5% LT (IL‐2: *Z* = −2.04, *P* < 0.05; IFN‐*γ*:* Z* = −2.40, *P* < 0.05).

## Discussion

The primary aim of this study was to determine whether CMV infection affects the exercise‐induced redeployment of T‐cell subsets following exercise of different intensities. We found that the effects of CMV infection and intensity on the numbers of T‐cell subsets in the blood after exercise were largely independent of each other. In both CMV+ and CMV− individuals, the greater exercise intensities resulted in significantly greater mobilizations of the T‐cell subsets to and from the blood compartment. Regardless of the exercise intensity, CMV+ subjects had a greater number of high‐differentiated CD4^+^ T cells and high‐differentiated CD8^+^ T cells compared to CMV−, and exhibited an enhanced mobilization of high‐differentiated T cells at all intensities.

Harboring a latent CMV infection alters the immune profile, leading to the expansion of populations of high‐differentiated T cells even at a relatively young age (Ouyang et al. 2003a; van de Berg et al. 2010). Because high‐differentiated T cells are mobilized by exercise to a greater extent than low‐differentiated T cells (Simpson et al. 2007, 2008; Campbell et al. 2009), it follows that CMV infection can augment the T‐cell response to exercise. Thus, we and others have reported an amplified mobilization of CD8^+^ T cells into and out of the peripheral blood with acute dynamic exercise among CMV+ individuals, driven by the greater number of high‐differentiated CD8^+^ T cells in CMV+ (Turner et al. 2010; Lavoy et al. 2014; Spielmann et al. 2014). A significantly greater exercise‐induced redeployment of high‐differentiated CD4^+^ T cells among CMV+ has also been demonstrated (Lavoy et al. 2014). In agreement with these earlier reports, this study found a significantly greater number of high‐differentiated CD4^+^ and CD8^+^ T cells in CMV+ compared to CMV−, as well as significant interaction effects between exercise and CMV on high‐differentiated CD4^+^ and CD8^+^ T cells. Unique to this study is the observation that CMV+ had a smaller ingress and egress of medium‐differentiated CD4^+^ T cells, and a smaller ingress of low‐differentiated CD8^+^ T cells after exercise compared to CMV−.

Differences in the mobilization of NK cells by exercise between CMV+ and CMV− have also been reported (Bigley et al. 2012, 2015). Bigley et al. (2015) demonstrated that the diminished responsiveness of NK cells to exercise in CMV+ is only observed following exercise intensities above lactate threshold. As CMV+ and CMV− exhibit similar increases in the catecholamines epinephrine and norepinephrine due to exercise, Bigley et al. (2015) explained the intensity‐dependent differential effect of CMV by demonstrating that NK cells in CMV+ exhibit impaired *β*‐adrenergic signaling. In contrast, we demonstrate here that the effects of CMV infection on the redeployment of high‐differentiated T‐cell subsets following exercise occur at intensities both above and below lactate threshold. Although not directly measured in this study, the lack of an intensity effect suggests that the high‐differentiated T cells of CMV+ and CMV−, unlike NK cells, do not differ in *β*‐adrenergic signaling pathways. The impact of CMV infection on NK‐cell and T‐cell function appears to be divergent, with largely negative effects on the T‐cell compartment (functional exhaustion and replicative senescence) but positive effects on the NK cells (increased cytotoxicity) (Bigley et al. 2016). The current results further support the notion of disparate effects of CMV on NK cells and T cells, with diverse mechanisms guiding its effect on cell redistribution with exercise.

A secondary aim of this study was to compare the expression of type I and type II cytokines by CD8^+^ T cells following exercise of different intensities. It is thought that exercise shifts the balance of type I and type II cytokine expression, with implications for altered immunity following exercise (Steensberg et al. 2001; Lancaster et al. 2004; Zhao et al. 2012). For example, both exhaustive cycling exercise and prolonged running exercise transiently decrease the number of IFN‐*γ* ^+^ T cells in circulation without changing the number of IL‐4^+^ T cells, thereby lowering the ratio of type I: type II cytokine^+^ cells (Steensberg et al. 2001; Lancaster et al. 2004). However, exercise has also been found to transiently increase the T‐cell expression of both type I and type II cytokines (Zaldivar et al. 2006; Lavoy et al. 2013). One methodological difference between these studies is the intensity of the exercise bout, as the latter employed shorter (Zaldivar et al. 2006) and less intense (Lavoy et al. 2013) exercise. We attempted to address this issue in this study by comparing three intensities of exercise within the same group of participants. We found a significant increase in the number of CD8^+^ T‐cells‐expressing type I and type II cytokines in the peripheral blood immediately following all intensities of exercise. Our results are similar to those of Lavoy et al. (2013) and Zaldivar et al. (2006), suggesting that the decreases in type I cytokines following exercise (as reported in (Steensberg et al. 2001; Lancaster et al. 2004)) are a result of prolonged exercise, rather than high‐intensity exercise.

There was a significant effect of exercise intensity on CD8^+^ T‐cell expression of type I cytokines, but not type II cytokines. The largest postexercise increase in IFN‐*γ* ^+^ CD8^+^ T cells occurred following the greatest exercise intensity, and the largest decrease 1‐h postexercise in IL‐2^+^ and IFN‐*γ* ^+^ CD8^+^ T cells was also observed following the greatest exercise intensity bout. The lack of responsiveness of type II cytokine expression to changes in intensity indicates the exercise‐induced changes in proinflammatory and anti‐inflammatory mediators follow different pathways. The lack of sensitivity of the type II cytokine IL‐4 to exercise intensity also led to an increase in the ratio of IFN‐*γ* ^+^: IL‐4^+^ T cells immediately following the +15% exercise bout, as the increased expression of IFN‐*γ* ^+^ by CD8^+^ T cells exceeded the increase in IL‐4 expression. The increased ratio suggests a transient shift in CD8^+^ T cells toward a more cytotoxic phenotype.

We have shown previously that increased T‐cell cytokine expression following exercise occurs mostly among CD27^−^ T cells (Lavoy et al. 2013). As CMV+ have a greater number of these cells, including after exercise, it follows that there may also be differences due to CMV in T‐cell cytokine expression following exercise. Furthermore, the high‐differentiated cells mobilized by exercise in CMV+ individuals have been shown to be highly functional cells, with a broad epitope diversity and the capacity for massive clonal expansion in response to CMV peptide stimulation in vitro (Spielmann et al. 2014). Unfortunately, we did not have the statistical power to compare cytokine responses between the CMV+ and CMV− participants using the nonparametric statistical tests that were required for cytokine analyses. Other limitations of the current project are that we cannot directly compare the present results to studies which have employed longer exercise sessions. We chose to use workloads that corresponded to percentages of the individuals' LT as realistic training zones typically used by competitive cyclists (Bourdon 2013). A relatively short bout (30 min) of exercise was selected to ensure that all participants could complete the highest intensity of exercise. We also cannot directly compare our results with others who have reported exercise‐induced changes in systemic cytokines, as changes in circulating cytokines may result from the activity of other immune cells not investigated here (e.g., monocyte) and other tissues (e.g., muscle) (Walsh et al. 2011). Finally, it should be noted that the participants in this study were trained cyclists, and that differences in the magnitude of the immune response to exercise (both T‐cell mobilization and cytokine expression) may have been found in untrained individuals.

In conclusion, this study presents two main findings. First, it was demonstrated that the impact of CMV infection on T‐cell redeployment following acute exercise is not dependent on the intensity of the exercise bout. CMV+ participants exhibited an augmented mobilization of high‐differentiated T cells compared to CMV− participants following exercise above and below lactate threshold. Second, all exercise intensities transiently increased CD8^+^ T‐cell expression of type I (IL‐2, IFN‐*γ*) and type II (IL‐4, IL‐10) cytokines. In addition, the magnitude of changes in IL‐2 and IFN‐*γ* expression by the CD8^+^ T cells was directly related to the intensity of exercise. Finally, the balance of IFN‐*γ* ^+^: IL‐4^+^ T cells was significantly increased following the greatest intensity of exercise, but returned to baseline value during recovery, suggesting a temporary enhancement of CD8^+^ T‐cell cytotoxicity. These results add to the newly developing body of literature examining the impact of infection history on the immune response to exercise.

## Conflict of Interest

The authors report no conflicts of interest.
